# Loss of prion protein induces a primed state of type I interferon-responsive genes

**DOI:** 10.1371/journal.pone.0179881

**Published:** 2017-06-26

**Authors:** Giulia Malachin, Malin R. Reiten, Øyvind Salvesen, Håvard Aanes, Jorke H. Kamstra, Kerstin Skovgaard, Peter M. H. Heegaard, Cecilie Ersdal, Arild Espenes, Michael A. Tranulis, Maren K. Bakkebø

**Affiliations:** 1 Faculty of Veterinary Medicine, Norwegian University of Life Sciences, Oslo, Norway; 2 Department of Microbiology, Division of diagnostics and intervention, Institute of Clinical Medicine, Oslo University Hospital, Rikshospitalet, Oslo, Norway; 3 Innate Immunology Group, Section for Immunology and Vaccinology, National Veterinary Institute, Technical University of Denmark, Kgs. Lyngby, Denmark; INSERM, FRANCE

## Abstract

The cellular prion protein (PrP^C^) has been extensively studied because of its pivotal role in prion diseases; however, its functions remain incompletely understood. A unique line of goats has been identified that carries a nonsense mutation that abolishes synthesis of PrP^C^. In these animals, the PrP-encoding mRNA is rapidly degraded. Goats without PrP^C^ are valuable in re-addressing loss-of-function phenotypes observed in *Prnp* knockout mice. As PrP^C^ has been ascribed various roles in immune cells, we analyzed transcriptomic responses to loss of PrP^C^ in peripheral blood mononuclear cells (PBMCs) from normal goat kids (*n* = 8, *PRNP*^+/+^) and goat kids without PrP^C^ (*n* = 8, *PRNP*^Ter/Ter^) by mRNA sequencing. PBMCs normally express moderate levels of PrP^C^. The vast majority of genes were similarly expressed in the two groups. However, a curated list of 86 differentially expressed genes delineated the two genotypes. About 70% of these were classified as interferon-responsive genes. In goats without PrP^C^, the majority of type I interferon-responsive genes were in a primed, modestly upregulated state, with fold changes ranging from 1.4 to 3.7. Among these were *ISG15*, *DDX58* (RIG-1), *MX1*, *MX2*, *OAS1*, *OAS2 and DRAM1*, all of which have important roles in pathogen defense, cell proliferation, apoptosis, immunomodulation and DNA damage response. Our data suggest that PrP^C^ contributes to the fine-tuning of resting state PBMCs expression level of type I interferon-responsive genes. The molecular mechanism by which this is achieved will be an important topic for further research into PrP^C^ physiology.

## Introduction

The cellular prion protein (PrP^C^) can misfold into disease-provoking conformers (PrP scrapie; PrP^Sc^) that give rise to several neurodegenerative prion diseases, such as Creutzfeldt-Jakob disease (CJD) in humans, scrapie in sheep and goats, and bovine spongiform encephalopathy in cattle [1]. The seeding of PrP^Sc^ in brain tissue acts as a template for further misfolding of PrP^C^, ultimately leading to severe neurodegeneration and neuronal death [1].

PrP^C^ is abundant throughout the nervous system, and, albeit at lower levels, in most other tissues of the body [2]. The protein is conserved in mammalian species [3, 4] and expressed already during early embryonal stages [5]. It was therefore surprising that *Prnp*^0/0^ mice developed normally and revealed no major phenotypes besides being prion-disease resistant [6–8]. Interestingly, in four *Prnp*^0/0^ mouse models (*Ngsk*, *Rcm0*, *ZrchII*, and *Rikn*), ablation of the *Prnp* gene induced severe degeneration of cerebellar Purkinje neurons [9–12]. This was, however, subsequently shown to be caused by ectopic expression of the prion-like protein Doppel (*Dpl*) in the brain, as a side-effect of the transgenic protocols [10]. Two additional *Prnp*-ablated mouse lines (*ZrchI* and *Npu*) displayed no neurodegeneration [7, 8]. Furthermore, other experiments have shown that a polymorphism in another *Prnp* flanking gene, *Sirp-alpha*, could significantly influence the interpretation of data that concerns the roles for PrP^C^ in phagocytosis [13]. Despite these inherent challenges with *Prnp*-null models [14], collectively known as the flanking-gene problem, the *Prnp*^0/0^ lines have proven extremely valuable in exploring PrP^C^ physiology. They have provided clues regarding maintenance of axonal myelin [15–17], modulation of circadian rhythms [18], and neuronal excitability [19], in addition to protective roles in severe stress such as ischemia [20] and hypoxic brain damage [21].

A more general problem is the gap between mice and human physiologies [22–24]. The two species diverged about 65 million years ago, and differ substantially in both size and life span. Mice have evolved into short-lived animals relying on massive reproductive capacity, whereas humans reside at the other end of the spectrum, with low reproduction rates and life spans of approximately 80 years. This is of particular significance in modeling chronic human diseases that take decades to develop, and often involve subtle immunological imbalances [22]. In addition, translation to human medicine has proven challenging.

Recently, we identified what seems to be a unique line of dairy goats carrying a nonsense mutation that completely abolishes synthesis of PrP^C^ [25]. This spontaneous, non-transgenic model, is referred to as *PRNP*^Ter/Ter^. Approximately 10 percent of the Norwegian dairy goat population carries the mutated allele. These animals appear to have normal fertility and behavior in all aspects of standard husbandry. We have no data to suggest that they are over-represented in disease statistics or otherwise failing in production performance. Careful analysis of hematological and blood biochemical parameters, as well as basic immunological features, did not reveal any abnormalities [26]. It was, however, noted that goats without PrP^C^ had slightly elevated numbers of red blood cells, identical to an observation in transgenic cattle without PrP^C^ [27], suggesting that this is a true biological loss-of-function phenotype, at least in ruminants.

Peripheral blood mononuclear cells (PBMCs) express moderate, but dynamic, levels of PrP^C^ [28]. We observed that goats heterozygous for the mutation (*PRNP*^+/Ter^) express half the amount of cell surface PrP^C^ on PBMCs [26]; however, a 50 percent reduction in levels compared to PBMCs from *PRNP*^+/+^ goats did not stimulate compensatory expression from the normal allele. Intrigued by this, and the fact that many reports have pointed to putative functions for PrP^C^ in immune cells (reviewed in [29], [30, 31]), mRNA sequencing of PBMCs derived from normal goats and goats without PrP^C^ was performed. The main goal of this study was to evaluate whether the loss of PrP^C^ elicits a transcriptional response in PBMCs that could reveal biological processes involving PrP^C^. Our findings show that in the absence of PrP^C^, a subtle, but highly significant change in the transcriptional profile of PBMCs is seen, dominated by upregulation in the expression of type I interferon-responsive genes.

## Results

### RNA-seq data quality control

**Fig 1:**
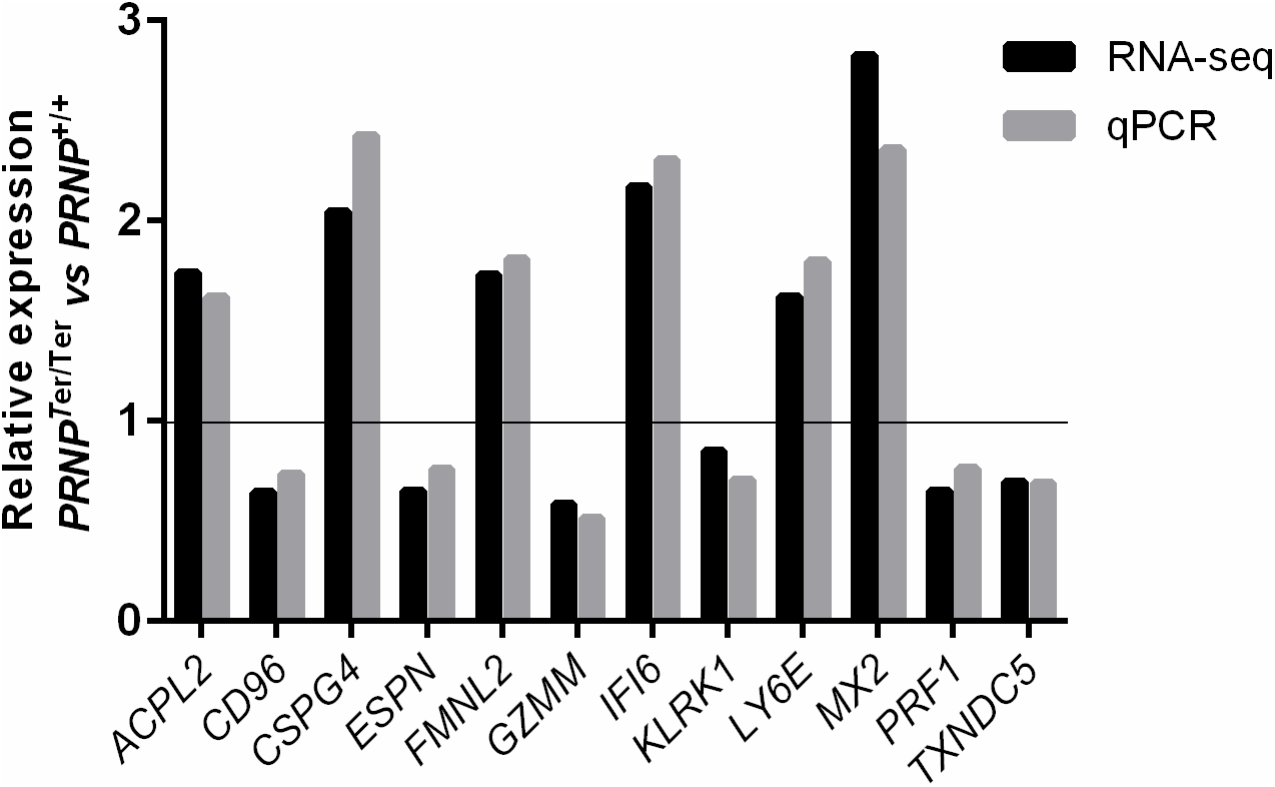
Validation of RNA sequencing data with quantitative PCR. Validation of 12 randomly chosen, differentially expressed genes was performed with qPCR using the original RNA. Expression data from the two methods are presented as relative expression between *PRNP*^Ter/Ter^ and *PRNP*^+/+^ animals (RNA-seq data *n* = 8, qPCR *n* = 6; r = 0.9616, p < 0.0001, Pearson correlation).

High quality RNA sequencing data (FASTQ) were derived from Beijing Genome Institute (BGI), with an average total reads of 58,806,319 per sample, average total mapped reads of 42,168,758, and average uniquely mapped reads of 38,253,898 per sample (S1 Fig). To validate the sequencing data, primers (S1 Table) were designed for 12 randomly selected differentially expressed genes (DEGs), using reverse transcription (RT) quantitative real-time PCR (qPCR) on the original RNA. As shown in Fig 1, qPCR analysis of mRNA levels correlated well with the RNA-seq analysis (r = 0.9616, p < 0.0001, Pearson correlation). Minor discrepancies could be due to sample variations, as RNA from only six goats per group were used for qPCR validation, compared with eight goats per group for RNA-seq analysis.

### Lack of PrP^C^ subtly alters the transcriptome in immune cells

A high correlation was observed between averaged *PRNP*^+/+^ and *PRNP*^Ter/Ter^ normalized gene expression data (r = 0.99, Pearson correlation). However, we found that not all *PRNP*^+/+^ and *PRNP*^Ter/Ter^ goats could be clearly separated from each other, probably reflecting the phenotypic diversity of the goats (S2 Fig). Despite this, using edgeR [32] and a p-value cut-off < 0.05, 735 genes were differentially expressed between the two genotypes (S1 File). Further filtration of the gene list using cut offs for fold change (log2 FC ± 0.5) and mean number of reads (> 100 reads in one of the groups) generated a high-confidence gene list of 127 DEGs, of which 67 were upregulated and 60 were downregulated in the *PRNP*^Ter/Ter^ genotype (S2 Table). Of note, as we have previously shown that the PBMC cell populations, mainly T cells, B cells and monocytes, are stable between the two genotypes compared in our study [26], the DEGs result from real genotype-associated shifts in gene expression, not shifts in the cell populations. Reassuringly, the *PRNP* gene was among the DEGs, with very few reads mapping to this locus in the mutant. The chromosomal distribution of the DEGs is found in S3 Fig. The *PRNP* gene is located on chromosome 13 in goats. Only 1 (*SIGLEC1*) of the 86 annotated DEGs also maps to chromosome 13. This gene is expressed at a low level and is irrelevant for the findings in our study.

**Fig 2:**
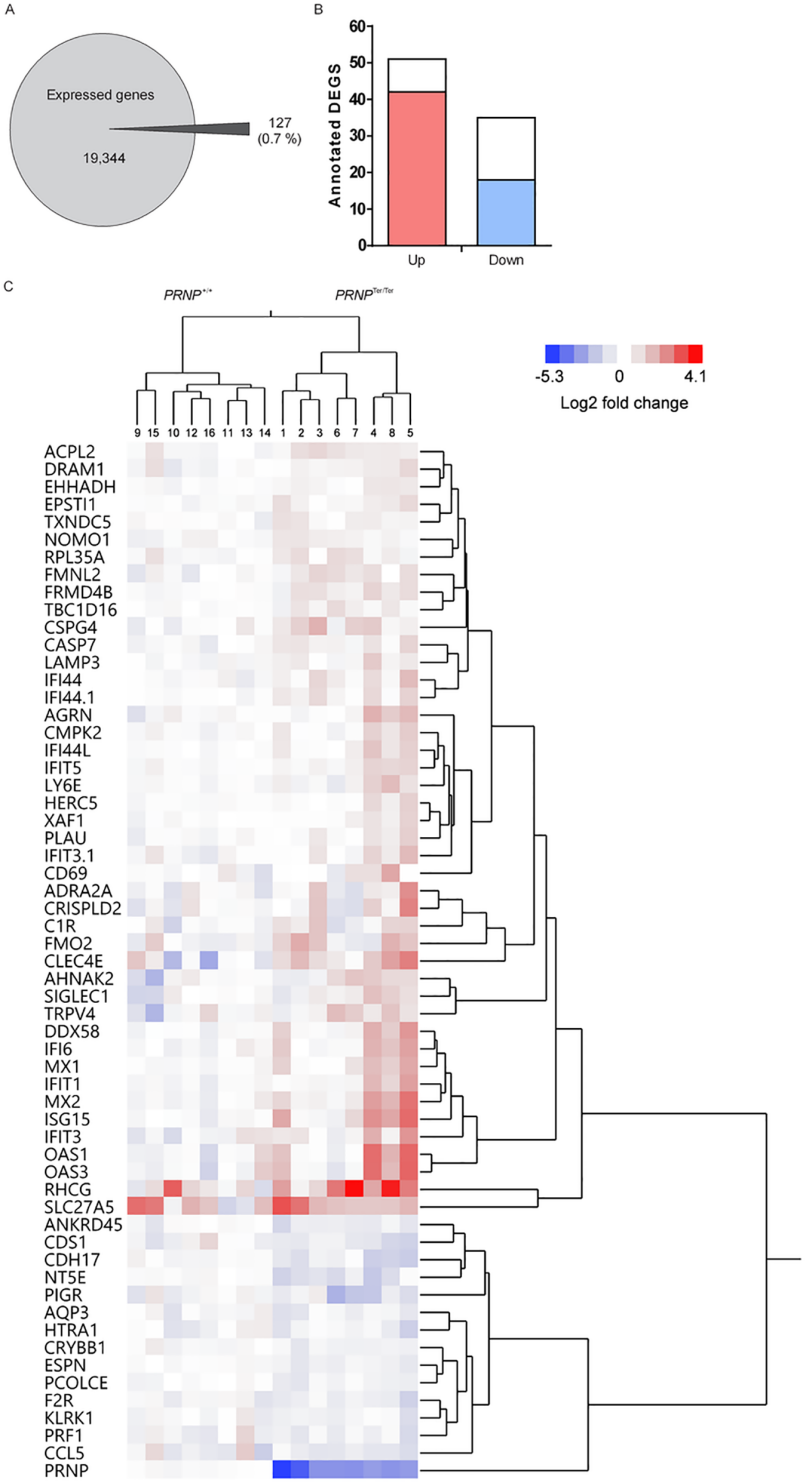
Interferon-responsive genes dominate among the differentially expressed genes in goats lacking PrP^C^. Graphical presentation of (A) the total number and percentage of differentially expressed genes (DEGs) between the two genotypes, compared to the average total number of genes expressed in peripheral blood mononuclear cells from both genotypes, and (B) the total number of upregulated and downregulated annotated DEGs. The fraction of upregulated (red) and downregulated (blue) interferon-responsive genes among the DEGs are also shown. (C) Hierarchical clustering of the interferon-responsive genes among the DEGs and expression data from all individual goats of both genotypes. Hierarchical clustering was performed using the ward algorithm on log2-normalized fold changes.

Of the average total number of genes expressed in PBMCs from both genotypes, only 0.7 percent of the genes were altered upon loss of PrP^C^ (Fig 2A). Using Ingenuity Pathway Analysis (IPA), of the 127 high-confidence DEGs, 86 genes were functionally annotated. Interestingly, 22 of these genes were categorized as “Viral infection” (p-value = 3.27x10^-5^), and additional genes were related to other anti-virus-associated terms. The majority of these genes were upregulated in the *PRNP*^Ter/Ter^ genotype compared with the *PRNP*^+/+^ genotype. Of the top canonical pathways, “Interferon signaling” was by far the most affected (p-value = 8.92x10^-6^). Due to these findings, we performed further analyses of the annotated DEGs using the Interferome database [33]. Strikingly, 60 of the 86 annotated DEGs were interferon-responsive genes (Fig 2B). Of these, 42 were upregulated (red bar) and 18 downregulated (blue bar) in the *PRNP*^Ter/Ter^ genotype. Fig 2C shows the inter-individual variation in gene expression of all samples represented in a heatmap, and hierarchical clustering analysis of the 60 interferon-responsive genes revealed a clustering of downregulated and upregulated genes between the *PRNP*^+/+^ and *PRNP*^Ter/Ter^ genotypes.

Since the observed data could be due to altered expression levels of interferons or components in type I interferon signaling, we analyzed expression levels of a number of genes that could affect the expression of interferon-responsive genes. However, differences between the genotypes were not detected (Table 1), except for *IFNB2-like*, which was slightly downregulated in the *PRNP*^Ter/Ter^ genotype (p-value = 0.025).

**Table 1:** Mean unique reads of genes related to Interferon signaling from *PRNP*^+/+^ (*n* = 8, ± SEM) and *PRNP*^Ter/Ter^ (*n* = 8, ± SEM) goats.

Gene symbol	Transcript ID	*PRNP*^+/+^	*PRNP*^Ter/Ter^
Interferons			
*IFNA-H-like*	XM_005683618.1	0.3 ± 0.3	0.0 ± 0.0
*IFNB2-like*	XM_005702021.1	63.0 ± 5.3	43.4 ± 4.2 *
*IFNK*	XM_005683589.1	0.1 ± 0.1	0.0 ± 0.0
*IFNO1-like*	XM_005683620.1	26.5 ± 6.8	19.1 ± 4.9
*IFNT2A*	XM_005683606.1	0.9 ± 0.4	0.9 ± 0.5
*IFNG*	XM_005680208.1	38.4 ± 10.9	27.8 ± 4.5
*IFNL3*	XM_005692539.1	0.1 ± 0.1	0.0 ± 0.0
*IFNL4-like*	XM_005692540.1	0.5 ± 0.3	0.3 ± 0.2
Interferon receptors			
*IFNAR1*	XM_005674742.1	11565.5 ± 613.5	11818.3 ± 683.0
*IFNAR2*	XM_005674684.1	3484.1 ± 245.4	3664.9 ± 188.7
*IFNGR1*	XM_005684807.1	3056.4 ± 268.9	3772.4 ± 252.6
*IFNGR2*	XM_005674741.1	7492.8 ± 179.1	8209.5 ± 408.9
*IFNLR1*	XM_005677011.1	95.5 ± 10.8	122.4 ± 22.4
Interferon signaling components			
*JAK1*	XM_005678310.1	31579.9 ± 920.9	31909.0 ± 908.7
*JAK2*	XM_005683698.1	2399.1 ± 109.3	2587.5 ± 84.7
*JAK3*	XM_005682189.1	11636.9 ± 600.5	9816.8 ± 603.9
*TYK2*	XM_005682457.1	4528.3 ± 205.6	4775.3 ± 328.4
*STAT1*	XM_005676277.1	26477.4 ± 2414.9	28314.6 ± 1119.4
*STAT2*	XM_005680347.1	5548.9 ± 332.1	6363.6 ± 408.4
*STAT3*	XM_005693850.1	98.5 ± 10.5	92.5 ± 8.2
*STAT4*	XM_005676278.1	2101.9 ± 158.6	1949.5 ± 120.6
*STAT5A*	XM_005693847.1	5250.6 ± 172.7	5365.3 ± 194.9
*STAT5B*	XM_005693846.1	4604.1 ± 137.3	4511.0 ± 155.2
*STAT6*	XM_005680308.1	15197.3 ± 704.8	15596.6 ± 692.7
*IRF1*	XM_005682621.1	12308.6 ± 1155.2	10936.5 ± 1329.5
*IRF2*	XM_005698710.1	624.5 ± 26.6	663.4 ± 17.1
*IRF3*	XM_005692726.1	1073.9 ± 74.3	1169.1 ± 60.4
*IRF4*	XM_005696935.1	1482.5 ± 157.3	1379.5 ± 140.9
*IRF5*	XM_005679456.1	764.9 ± 61.4	811.8 ± 65.6
*IRF6*	XM_005691036.1	7.3 ± 2.2	5.4 ± 1.9
*IRF8*	XM_005691907.1	3565.8 ± 219.0	3824.8 ± 210.8
*IRF9*	XM_005685224.1	205.8 ± 16.7	234.5 ± 28.2
Inhibitors and enhancers			
*IRF2BP-like*	XM_005686182.1	2686.3 ± 135.5	2794.5 ± 123.7
*IRF2BP1*	XM_005692789.1	1265.3 ± 33.0	1232.4 ± 32.3
*IRF2BP2*	XM_005699013.1	8090.8 ± 600.5	8588.9 ± 793.4
*PIAS1*	XM_005685148.1	1266.8 ± 66.9	1320.0 ± 86.4
*PIAS2*	XM_005697179.1	390.6 ± 16.9	405.0 ± 19.2
*PIAS3*	XM_005677741.1	99.1 ± 7.8	99.5 ± 8.1
*PIAS4*	XM_005682570.1	39.4 ± 2.7	40.0 ± 4.8
*SOCS2*	XM_005679820.1	0.8 ± 0.4	1.4 ± 0.6
*SOCS3*	XM_005694412.1	372.0 ± 48.8	346.4 ± 47.5
*SOCS4*	XM_005685884.1	845.4 ± 24.5	823.3 ± 28.0
*SOCS5*	XM_005686570.1	1748.9 ± 82.0	1737.6 ± 75.8
*SOCS6*	XM_005709580.1	137.5 ± 14.4	144.6 ± 14.0
*SOCS7*	XM_005709575.1	2286.3 ± 193.8	2144.5 ± 198.7
*IL18*	XM_005689450.1	21.3 ± 4.5	18.9 ± 3.6
*PTK2*	XM_005688815.1	82.4 ± 11.4	92.1 ± 7.4
*PTK2B*	XM_005684041.1	99.3 ± 11.6	114.5 ± 17.5

*: p = 0.025

### Introduction of *PRNP* inhibited *MX2* gene expression in SH-SY5Y cells

**Fig 3:**
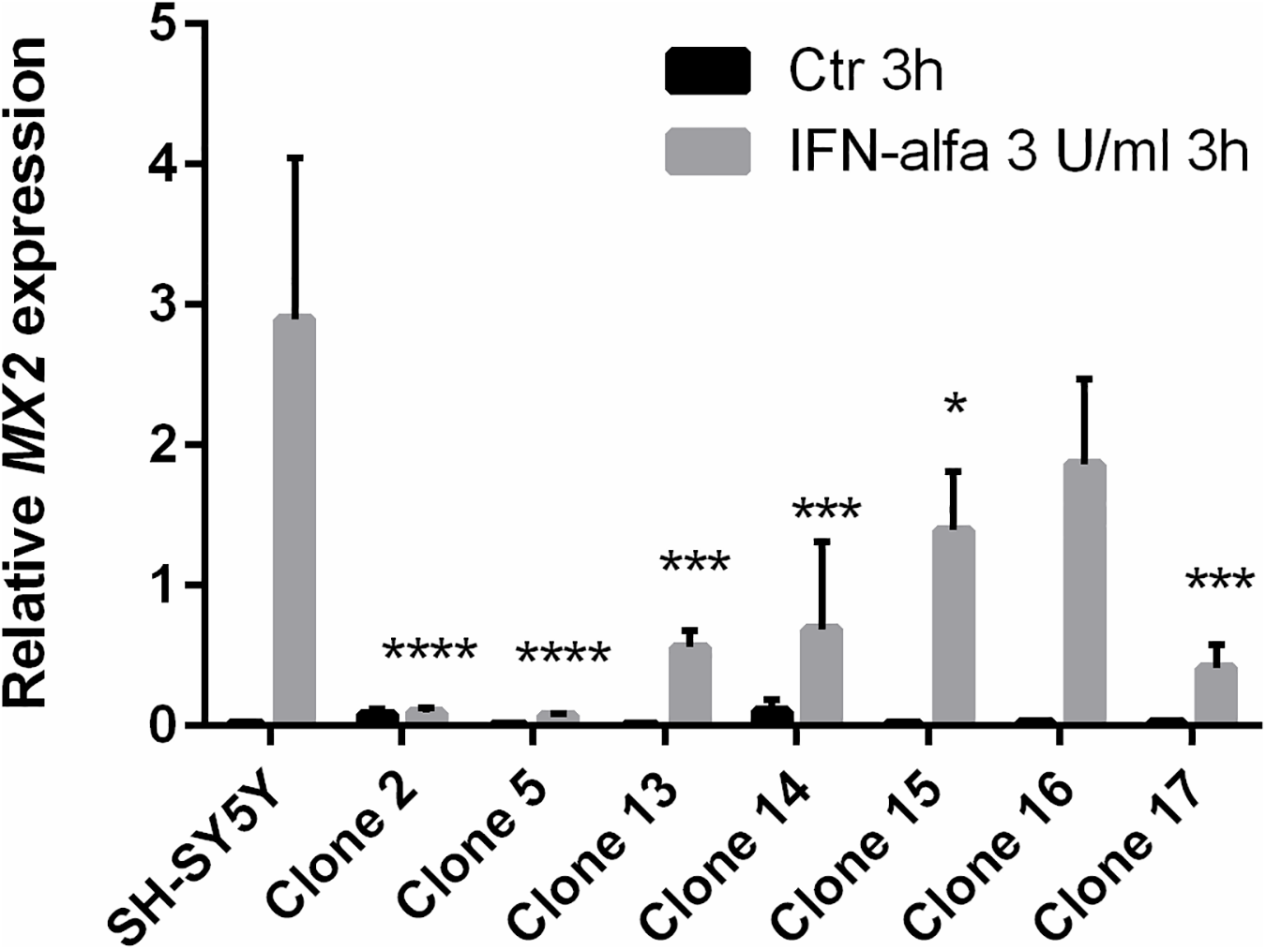
PrP^C^ suppresses upregulation of *MX2* gene expression upon INF-α stimulation in SH-SY5Y cells. Untransfected human neuroblastoma SH-SY5Y cells and seven different clones transfected with a plasmid containing human *PRNP* to produce SH-SY5Y clones expressing human PrP^C^, were stimulated for 3h with IFN-α (3 U/ml) (mean ± SEM, *n* = 4), and *MX2* gene expression was assessed. Six out of seven clones displayed a significantly lower response to IFN-α compared with the untransfected SH-SY5Y cells, using Dunnett’s post hoc test for multiple comparisons.

To test whether PrP^C^ could influence IFN-α responsiveness in a cell culture system with a different genetic makeup, we used human neuroblastoma SH-SY5Y cells, which normally express extremely low levels of PrP^C^. SH-SY5Y clones stably expressing human PrP^C^ were generated (SH-SY5Y PrP^high^) and assessed with regard to glycosylation and proteolytic processing to ensure physiological post-translational modification and trafficking of PrP^C^ (S4 Fig). Eight clones stably expressing PrP^C^ as well as untransfected SH-SY5Y cells were exposed to 3 U/ml IFN-α for 3h. One of the transfected clones showed aberrantly high *MX2* gene expression levels and was excluded from the analysis. Of the seven clones included in the experiment, six displayed a significantly reduced response to IFN- α, as assessed by the interferon-responsive gene *MX2* expression levels, compared with the untransfected SH-SY5Y cells, using Dunnett’s post hoc test for multiple comparisons (Fig 3) (*n* = 4, mean ± SEM). The levels of PrP^C^ expression did not directly correlate with the degree of *MX2* expression-level inhibition; however, this was not expected due to the complexity of the interferon signaling pathway, and the possible distance between PrP^C^ interference and *MX2* gene expression. On average, the clones showed a significantly inhibited response to IFN- α (p-value = 0.0001) compared with the untransfected SH-SY5Y cells, using a two-way ANOVA.

### Increased interferon-responsive gene expression in blood leukocytes devoid of PrP^C^ after LPS challenge

**Fig 4:**
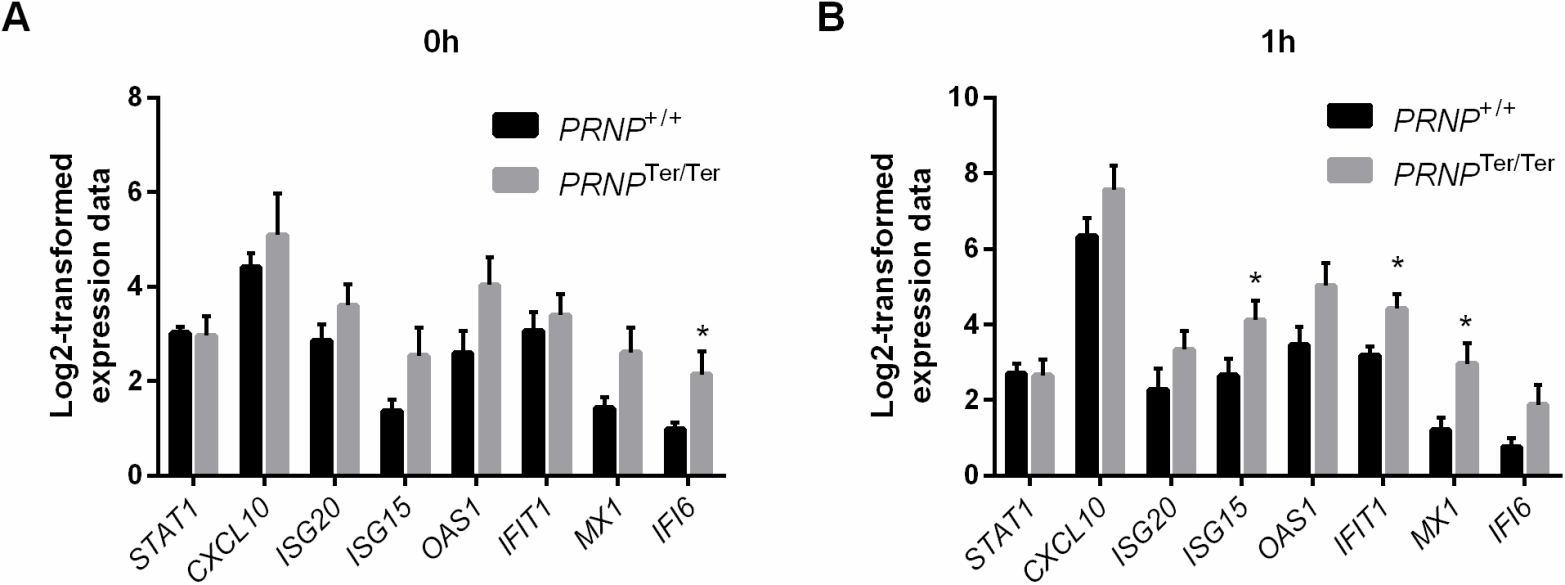
Expression of interferon-responsive genes in blood leukocytes after *in vivo* lipopolysaccharide (LPS) challenge in goats without PrP^C^. RNA was extracted from circulating blood leukocytes from both genotypes, and gene expression was analyzed by FLUIDIGM qPCR. (A) Basal expression level (0 h) of selected interferon-responsive genes and *STAT1* in *PRNP*^+/+^ (*n* = 12) and *PRNP*^Ter/Ter^ (*n* = 13) animals. (B) Gene expression of interferon-responsive genes and *STAT1* after *in vivo* LPS challenge (1 h) from *PRNP*^+/+^ (*n* = 7) and *PRNP*^Ter/Ter^ (*n* = 8) animals. Values are mean ± SEM. Statistical significance is indicated by *, p-value < 0.05, as assessed by multiple t-tests.

In an independent, parallel study [34, 35], goats were challenged intravenously with lipopolysaccharide (LPS), thereby indirectly stimulating interferon pathways. RNA was extracted from circulating blood leukocytes, and gene expression of interferon-responsive genes was assessed by FLUIDIGM qPCR. As shown in Fig 4A, basal level expression (0h) of several interferon-responsive genes was slightly higher in the *PRNP*^Ter/Ter^ (*n* = 13) genotype than in the *PRNP*^+/+^ (*n* = 12) genotype, albeit being significantly different for only *IFI6* (p-value = 0.037). Moreover, *STAT1* mRNA expression levels did not differ between the genotypes. One hour after LPS challenge, the mRNA expression level of interferon-responsive genes increased slightly and the difference between the two genotypes was more pronounced (Fig 4B), with three genes showing a statistically significant difference in expression level (*ISG15* (p-value = 0.049), *IFIT1* (p-value = 0.02), and *MX1* (p-value = 0.019), assessed by multiple t-tests).

## Discussion

Similar to observations in transgenic mice [6], goats [36], and cattle [27] with knockout (KO) of *PRNP*, the *PRNP*^Ter/Ter^ goats display no obvious loss-of-function phenotype [25, 26]. Consequently, only subtle transcriptomic alterations were expected, corroborating data from KO mouse models [37–41]. Accordingly, this study revealed subtle expression differences affecting less than a percent of the expressed genes. However, analysis of the annotated DEGs using the Interferome database [33], identified a distinct expression profile, with 70 percent of the DEGs being classified as interferon responsive, of which several were among the top upregulated genes. Importantly, animals were age-matched and derived from the same research flock. The health status of this herd is frequently monitored and considered excellent. Prior to sampling, animals were assessed clinically by a veterinarian and found healthy, which was also confirmed by hematological analysis in an accompanying study [26]. Furthermore, we were unable to detect any differences in gene expression levels of neither interferons nor IFN signaling components. A flanking gene problem will also be present in the *PRNP*^Ter/Ter^ goats; however, preliminary data indicate that this is very limited compared to inbred knockout mouse models. In the absence of alternative explanations, we consider the observed gene expression profile to be a true signature of PrP^C^ loss-of-function. It is likely that this profile, which is evident at rest in the outbred and immunocompetent goats, might be even weaker or absent in inbred transgenic mice, housed in pathogen-depleted environments. It is, however, interesting to note that studies of prion disease in mice have revealed a gene expression profile similar to that observed in PrP^C^-deficient goats. Analysis of transcripts from mouse whole brain throughout the course of experimental CJD revealed an upregulation of several interferon-responsive genes, e.g. *OAS*, *ISG15*, and *IRF*-family members. Importantly, the upregulation of these genes occurred very early in the course of the disease, approximately 50 days before the onset of neuropathological signs and detection of PrP^Sc^ [42]. Similar findings were recently reported in another study of prion-infected mice [43]. In a hamster model of scrapie, several interferon-responsive genes, including those encoding OAS and Mx protein, were upregulated during development of scrapie [44]. In addition, three interferon-responsive genes, assessed by qPCR studies, were moderately upregulated in a hamster model and different mouse models inoculated with scrapie strains [45]. Recently, transcriptomic data from cerebellar organotypic cultured slices infected with prions showed that a slight upregulation of several interferon-responsive genes was evident at 38 and 45 days post infection [46]. It is tempting to speculate that some of the observed gene expression alterations at very early stages of prion disease could, at least partly, reflect induced loss-of-PrP^C^ function, and, thus, explain the similarity with the expression profile reported here. Further investigations are clearly needed to test this hypothesis.

Studies of human SH-SY5Y neuroblastoma cells transfected with human *PRNP* displayed a significantly dampened response (MX2 expression) to a low-level IFN-α stimulation, compared with untransfected cells that are virtually devoid of PrP^C^. Furthermore, in an independent, parallel study involving older goat kids than those recruited for the RNA seq study, animals were challenged with LPS, which is a potent pro-inflammatory compound. In contrast to mice, which are relatively tolerant towards LPS, goats have a similar sensitivity as humans [34, 35]. In line with data from the present RNA sequencing study, resting state expression levels of interferon-responsive genes in leukocytes were slightly elevated in the *PRNP*^Ter/Ter^ genotype. Interestingly, the expression differences between the genotypes were increased one hour after LPS injection. Apparently, leukocytes without the expression of PrP^C^ upregulated interferon-responsive genes more rapidly than their PrP^C^-expressing counterparts. The regulation of interferon-responsive genes expression level is multifaceted and tightly controlled at several levels [47, 48], involving receptor downregulation, upregulation of a plethora of inhibitors as well as epigenetic modifications.

Taken together, our data suggest that PrP^C^ contributes to dampening of type I interferon signaling at rest and that loss of PrP^C^ induces a primed state of interferon-responsive genes. Accordingly, direct or indirect stimulation of type I IFN signaling, elicits a somewhat stronger immediate response when PrP^C^ is absent. These data do not conflict with roles acclaimed to the prion protein. Indeed, they might strengthen previous observations and provide mechanistic hints of PrP^C^ physiology.

## Material and methods

### Animals

The animals (FOTS approval number ID 8058) included in the study were of the Norwegian Dairy Goat Breed obtained from a research herd of approximately 100 winter-fed goats at the Norwegian University of Life Sciences. Based on health surveillance through membership in the Goat health monitoring service and The Norwegian Association of Sheep and Goat Farmers and daily monitoring, the general health status of the herd is considered to be good. The entire flock was previously genotyped [25] concerning *PRNP* genotypes, and through selective breeding, goat kids with the two genotypes *PRNP*^+/+^ (*n* = 8; 4 female and 4 male) and *PRNP*^Ter/Ter^ (*n* = 8; 4 female and 4 male) were retrieved. Prior to inclusion in the experiment, all goat kids were examined clinically and found to be healthy.

### Isolation of peripheral blood mononuclear cells

Blood was sampled from the jugular vein into EDTA tubes at 2–3 months of age. Peripheral blood mononuclear cells (PBMCs) were isolated by gradient centrifugation (Lymphoprep®, Axis-Shield, Dundee, Scotland) at 1760 x *g* without brake, and washed with PBS supplemented with EDTA (2 mM). Red blood cells were lysed by brief exposure to sterile water, and washed with PBS supplemented with EDTA (2 mM) prior to counting and trypan blue viability assessment using a Countess® Automated Cell Counter (Life Technologies, Thermo Fisher Scientific, Waltham, MA).

### Cell culture studies

Human neuroblastoma SH-SY5Y cells (Sigma-Aldrich, Merck, Kenilworth, NJ) were cultured in Eagle’s Minimum Essential Medium and Ham’s F12 (1:1) (Sigma-Aldrich) supplemented with 10% heat-inactivated fetal bovine serum (FBS), glutamine and antibiotics (1% streptomycin and penicillin) (all from Gibco, Thermo Fisher Scientific), and cultivated in T25 flasks at 37°C with 5% (v/v) CO_2_ at saturated humidity. SH-SY5Y cells were stably transfected with a plasmid construct, pCI-neo (Promega, Madison, WI) encoding human *PRNP*, using jetPRIME (Polyplus, Illkirch, France) according to the manufacturer's instructions. Transfected cells were grown under selection pressure of Geneticin (Thermo Fisher Scientific), and nine different single clones with variable levels of PrP^C^ (SH-SY5Y PrP^high^) were isolated (S4 Fig). Clone no. 8 showed an abnormal phenotype, and was excluded from the studies.

### Western blotting

Untransfected SH-SY5Y cells and transfected SH-SY5Y PrP^high^ clones were lysed in homogenizer buffer (Tris HCl 50 uM, NaCl 150 mM, EDTA 1 mM, DOC 0.25%, NP40 1%) supplemented with protease inhibitor cocktail (Roche complete, Roche Holding AG, Basel, Switzerland). Protein concentrations were measured using Protein assay (Bio-Rad, Hercules, CA). To obtain deglycosylated protein, 20 μg of total protein were incubated overnight with PNGase-F (New England Biolabs, Ipswich, MA), according to the manufacturer’s instructions.

Fifty μg of protein or the deglycosylated samples were separated on sodium dodecyl sulfate (SDS) polyacrylamide gel electrophoresis (12% Criterion™ XT Bis-Tris, Bio-Rad), and transferred to polyvinylidene fluoride (PVDF) membranes (GE Healthcare, Little Chalfont, United Kingdom). After incubation with blocking buffer (5% non-fat milk in TBS-Tween) for 90 minutes at room temperature, samples were incubated in 1% non-fat milk in TBS-Tween containing mouse anti-PrP^C^ primary antibody diluted 1:4000 (6H4, Prionics, Thermo Fischer Scientific) over-night at 4°C. Subsequently, the membrane was washed and incubated for 90 minutes in 1% non-fat milk containing Alkaline Phosphatase (AP)-conjugated anti-mouse IgG diluted 1:4000 (Novex, Life Technologies, Thermo Fischer Scientific). Membrane was developed using EFC™ substrate (GE Healthcare) and visualized with Typhoon 9200 (Amersham Bioscience, GE Healthcare).

### Isolation and sequencing of RNA

Total RNA was extracted using the Qiagen RNeasy mini plus kit (Qiagen, Germantown, MD) following the manufacturer’s instructions. RNA concentration and purity was analyzed using NanoDrop-1000 Spectrophotometer (Thermo Fisher Scientific) or Epoch Microplate Spectrophotometer (BioTek Instruments Inc, Winooski, VT), and quality was assessed before RNA sequencing using RNA Nano Chips on an Agilent 2100 Bioanalyzer (both from Agilent Technologies, Santa Clara, CA). RNA was stored at -80°C. Individual RNA samples of high quality (RIN ≥ 9.8) were sequenced by mRNA poly-A-tail, paired-end sequencing (Illumina HiSeq 2000) with 91 bp read-lengths (Beijing Genomics Institute (BGI), Hong Kong), retrieving a minimum depth of 5G clean data per sample. In detail, after the total RNA extraction and DNase I treatment, magnetic beads with Oligo (dT) were used to isolate mRNA. Mixed with the fragmentation buffer, the mRNA was fragmented into short fragments, and cDNA was synthesized using the mRNA fragments as templates. Short fragments were purified and resolved with EB buffer for end reparation and single nucleotide A (adenine) addition. The short fragments were connected with adapters. After agarose gel electrophoresis, the suitable fragments were selected for the PCR amplification as templates. During the QC steps, Agilent 2100 Bioanalyzer and ABI StepOnePlus Real-Time PCR System were used in quantification and qualification of the sample library.

For the IFN-studies, RNA quality was assessed by TAE/formamide RNA gel electrophoresis. RNA samples were mixed with formamide (50% v/v, Sigma) and orange loading dye (New England Biolabs), denatured by heating for 5 min at 65°C, put on ice, and loaded on 1% agarose gel containing 1xTAE buffer (0.04 M Tris-acetate, 1 mM EDTA) and visualized with SYBR™ Safe (Invitrogen, Thermo Fisher Scientific).

### Analysis of RNA sequencing data

Reads were mapped to the goat genome assembly (CHIR_1.0) using SOAP2 [49]. Reads per gene were obtained using SOAP2 and the goat genome annotation (RefSeq, CHIR_1.0). Read counts were normalized to reads per kilobase per million mapped reads (RPKM) [50]. Testing for differentially expressed genes was performed using the function exactTest in edgeR [32].

### Expression analysis by reverse transcription (RT) quantitative real-time PCR (qPCR) analysis

cDNA was synthesized using SuperScript III Reverse Transcriptase, RNase Out, dNTP mix and Random Primers (all from Invitrogen, Thermo Fisher Scientific) at the following conditions: 5 min at 65°C, >1 min on ice, 5 min at 25°C, 1 h at 50°C and 15 min at 70°C.

For the RNA sequencing validation study, qPCR was conducted with LightCycler 480 Sybr Green I Master mix (Roche). cDNA corresponding to 2.5 ng RNA was used per reaction. The samples were run in duplicates in a total volume of 20 μl on a LightCycler 96 System (Roche). Conditions: 5 min at 95°C; 40 cycles of 10 sec at 95°C, 10 sec at 60°C and 10 sec at 72°C; and melting curve with 5 sec at 95°C, 1 min at 65°C and 97°C. Relative expression levels were calculated using a standard curve generated from one randomly selected animal, run in triplicate, with GAPDH as a reference gene, and one randomly selected animal as a positive control. The average of six *PRNP*^Ter/Ter^ animals was divided by the average of six *PRNP*^+/+^ animals, and compared relative to RNA sequencing data.

For the interferon-treatment studies using SH-SY5Y cells, qPCR was conducted with LightCycler 480 Sybr Green I Master mix (Roche). cDNA corresponding to 10 ng RNA was used per reaction. The samples were run in triplicate in a total volume of 10 μl on a LightCycler 96 System (Roche). Conditions: 5 min at 95°C; 40 cycles of 10 sec at 95°C, 10 sec at 60°C and 10 sec at 72°C; and melting curve with 5 sec at 95°C, 1 min at 65°C and 97°C. Relative expression levels were calculated using the ΔΔCt method. ActB was used as a reference gene. An inter-run calibrator was included on every plate as a positive control. The qPCR-amplified sample was run on a 1% agarose gel, and visualized using SYBR™ Safe (Thermo Fisher Scientific).

### LPS challenge and FLUIDIGM qPCR of whole blood leukocyte interferon-responsive genes

An intravenous LPS challenge was performed (0.1 μg/kg, *Escherichia coli* O26:B6) in 16 Norwegian dairy goats age 6–7 months (*8 PRNP*^*+/+*^ (female) and 8 *PRNP*^*Ter/*Ter^ (7 female, 1 castrated male)) (FOTS approval number IDs 5827, 6903, and 7881), and 10 controls were included (5 of each genotype). In brief, blood samples were collected in PAX-gene blood RNA tubes before (0 h) and after LPS challenge (1 h). High quality RNA (RIN 9.0 ± 0.34) was extracted using the PAXgene Blood miRNA kit, and cDNA synthesis was performed in two replicates (QuantiTect Reverse Transcription Kit). The relative expression of ISGs in circulating leukocytes was assessed after qPCR on the Fluidigm Biomark HD platform and data analysis using GenEx5 software (MultiD, Sweden). The full study protocol, method description, and primer sequences can be found in [34, 35].

### Statistical analysis

Multiple *t*-tests or two-way ANOVA followed by Dunnett’s post hoc test for multiple comparisons were used for statistical analysis of the data using Graph Pad Prism v. 6.07 (Graphpad, La Jolla, CA). For correlation analysis, the Pearson correlation coefficient was calculated. Mean values are presented ± SEM.

### Ethics statement

The animal experiments were performed in compliance with ethical guidelines, and approved by the Norwegian Animal Research Authority (FOTS approval number IDs 8058, 5827, 6903, and 7881) with reference to the Norwegian regulation on animal experimentation (FOR-2015-06-18-761).

## Supporting information

S1 Fig Individual number of reads obtained from RNA sequencing. Total reads, total mapped reads and uniquely mapped reads across all samples, *n* = 16, 8 of each genotype.
(TIF)

S2 Fig Hierarchical clustering dendrogram. Hierarchical clustering dendrogram of all genes after normalization of expression data (RPKM) using Euclidean distance and complete linkage.
(TIF)

S3 Fig Chromosomal distribution of differentially expressed genes. (A) Frequency of differentially expressed genes (735 genes) per chromosome. Total number of genes per chromosome were obtained from National Center for Biotechnology Information (NCBI), based on the Capra hircus CHIR_1.0-Primary Assembly. (B) Chromosomal distribution of annotated differentially expressed genes (86 genes).
(TIF)

S4 Fig Clones of human neuroblastoma SH-SY5Y cells expressing human *PRNP*. Protein expression of PrP^C^ for untreated and PNGase-F-treated untransfected human neuroblastoma SH-SY5Y cells and SH-SY5Y clones transfected with human *PRNP* (*n* = 8), determined by Western Blot analysis using 6H4 mouse anti-PrP^C^ as the primary antibody. Protein bands correspond to glycosylated PrP^C^, deglycosylated PrP^C^ and PrP^C^ C1 fragment as indicated.
(TIF)

S1 Table Forward and reverse primers used for qPCR. (DOCX)

S2 Table Differentially expressed genes between *PRNP*^Ter/Ter^ (*n* = 8) and *PRNP*^+/+^ (*n* = 8) goats (127 genes). (DOCX)

S1 File Differentially expressed genes (735 genes). (XLSX)
