# Supplementary material for: Loss of prion protein induces a primed state of type I interferon-responsive genes
Source: PLoS One. 2017 Jun 26;12(6):e0179881. doi: 10.1371/journal.pone.0179881 (PMC5484497; doi:10.1371/journal.pone.0179881)
Supplement: S1 Table — (DOCX) [file pone.0179881.s005.docx]

**S1 Table: Forward and reverse primers used for qPCR**

| Gene symbol | Species | Forward 5' to 3' | Reverse 5' to 3' |
| --- | --- | --- | --- |
| *ACPL2* | Goat | TTCTAAAAGGCGCACGATGC | TTCTGGCCTTTGCAAACCAC |
| *ActB* | Human | CCGCGAGAAGATGACCCAGAT | GGATAGCACAGCCTGGATAGCA |
| *CD96* | Goat | ATGCATTTGGTCAGGGGAGTAG | AGCCAGGCAAAGCGTAAATG |
| *CSPG4* | Goat | ATGTTCAGCGTCATCATCCC | TGCCTGTCTTGTTGCGTTTG |
| *ESPN* | Goat | AAAGTGCCCAAGCTGTTGAC | AGAGCTCTGCACACTTTGAC |
| *FMNL2* | Goat | TGCCGAAACCAAGAATGCAG | TGTGTCCTGCAGTTTTTCCG |
| *GAPDH* | Goat | GGTTGTCTCCTGCGACTTCA | TGGAAATGTGTGGAGGTCGG |
| *GZMM* | Goat | TGCACCGACATCTTCAAACC | GCTGCACAATGCTCCTTAGAAC |
| *IFI6* | Goat | TATCGCTGTTCCTGTGCTACC | AAGCTCGAGTCGCTGTTTTC |
| *KLRK1* | Goat | ATGGAACCTGTGCAGTCTATGG | TGGCGAATGGCTTTTGAGTC |
| *LY6E* | Goat | AAGCAAAGCAACTGGGACTG | CAAGTTCACCACGTTCTTGAGG |
| *MX2* | Goat | TTCACGGAAACCAGCAAACG | TGCATCATGGCTTTCTGCAC |
| *MX2* | Human | AGAGGCAGCGGAATCGTAAC | GGTGTTCCGGTAGCTGATCC |
| *PRF1* | Goat | ACCATCGTTCAAGGCATGTG | ACCATCGACATTGGAATGGC |
| *PRNP* | Goat | GTGGCTACATGCTGGGAAGT | AGCCTGGGATTCTCTCTGGT |
| *PRNP* | Human | CTGCTGGATGCTGGTTCTCT | GTGTTCCATCCTCCAGGCTT |
| *TXNDC5* | Goat | AAGTTTTACGCGCCATGGTG | AGTACTTGCTGCAGAGGTTCC |
