# Supplementary material for: Loss of prion protein induces a primed state of type I interferon-responsive genes
Source: PLoS One. 2017 Jun 26;12(6):e0179881. doi: 10.1371/journal.pone.0179881 (PMC5484497; doi:10.1371/journal.pone.0179881)
Supplement: S2 Table — (DOCX) [file pone.0179881.s006.docx]

**S2 Table: Differentially expressed genes between *PRNP*^Ter/Ter^ (*n* = 8) and *PRNP*^+/+^ (*n* = 8) goats**

| **Gene symbol*** | **Transcript ID** | **Log2FC** | **Ratio** | **Description** |
| --- | --- | --- | --- | --- |
| ***ACPL2*** | **XM_005675502.1** | **0,80** | **1,74** | **acid phosphatase-like 2** |
| ***ADAMDEC1*** | **XM_005684016.1** | **-1,32** | **0,40** | **ADAM-like, decysin 1** |
| ***ADRA2A*** | **XM_005698608.1** | **0,87** | **1,82** | **adrenoceptor alpha 2A** |
| ***AGRN*** | **XM_005690869.1** | **0,88** | **1,84** | **agrin** |
| ***AHNAK2*** | **XM_005695474.1** | **0,77** | **1,71** | **AHNAK nucleoprotein 2** |
| ***ANKRD45*** | **XM_005690886.1** | **-0,82** | **0,57** | **ankyrin repeat domain 45** |
| ***ANO9*** | **XM_005700107.1** | **-0,61** | **0,66** | **anoctamin 9** |
| ***AQP3*** | **XM_005684063.1** | **-0,59** | **0,67** | **quaporin 3 (Gill blood group)** |
| ***ATP8B3*** | **XM_005682922.1** | **0,73** | **1,66** | **ATPase, aminophospholipid transporter, class I, type 8B, member 3** |
| *BAC7.5* | XM_005696009.1 | -2,01 | 0,25 | Bac7.5 protein |
| ***C1R*** | **XM_005680921.1** | **0,73** | **1,66** | **complement component 1, r subcomponent, transcript variant X2** |
| *C3H1orf162* | XM_005677896.1 | -0,61 | 0,66 | chromosome 3 open reading frame, human C1orf162 |
| ***CASP7*** | **XM_005698497.1** | **0,65** | **1,57** | **caspase 7, apoptosis-related cysteine peptidase, transcript variant X1** |
| ***CCDC14*** | **XM_005675042.1** | **0,54** | **1,45** | **coiled-coil domain containing 14** |
| ***CCDC67*** | **XM_005699410.1** | **-0,69** | **0,62** | **coiled-coil domain containing 67** |
| ***CCDC8*** | **XM_005709596.1** | **-1,47** | **0,36** | **coiled-coil domain containing 8** |
| ***CCL5*** | **XM_005693201.1** | **-0,86** | **0,55** | **chemokine (C-C motif) ligand 5** |
| ***CD69*** | **XM_005680868.1** | **0,69** | **1,61** | **CD69 molecule** |
| ***CD96*** | **XM_005674903.1** | **-0,65** | **0,64** | **CD96 molecule** |
| ***CDH17*** | **XM_005689238.1** | **-0,96** | **0,51** | **cadherin 17, LI cadherin (liver-intestine)** |
| ***CDHR5*** | **XM_005700099.1** | **0,59** | **1,51** | **cadherin-related family member 5** |
| ***CDS1*** | **XM_005681958.1** | **-0,96** | **0,51** | **CDP-diacylglycerol synthase (phosphatidate cytidylyltransferase) 1** |
| ***CLEC4E*** | **XM_005680910.1** | **1,54** | **2,90** | **C-type lectin domain family 4, member E** |
| ***CLU*** | **XM_005683560.1** | **-0,72** | **0,61** | **clusterin** |
| ***CMPK2*** | **XM_005687096.1** | **0,60** | **1,51** | **cytidine monophosphate (UMP-CMP) kinase 2, mitochondrial** |
| ***CRISPLD2*** | **XM_005691843.1** | **0,93** | **1,91** | **cysteine-rich secretory protein LCCL domain containing 2** |
| ***CRYBB1*** | **XM_005691704.1** | **-0,54** | **0,69** | **crystallin, beta B1** |
| ***CSPG4*** | **XM_005695190.1** | **1,03** | **2,04** | **chondroitin sulfate proteoglycan 4** |
| ***DDX58*** | **XM_005683566.1** | **1,16** | **2,24** | **DEAD (Asp-Glu-Ala-Asp) box polypeptide 58** |
| ***DHDH*** | **XM_005692697.1** | **-0,77** | **0,59** | **dihydrodiol dehydrogenase (dimeric)** |
| ***DOCK6*** | **XM_005682467.1** | **0,83** | **1,77** | **dedicator of cytokinesis 6** |
| ***DRAM1*** | **XM_005680638.1** | **0,60** | **1,52** | **DNA-damage regulated autophagy modulator 1** |
| ***DTWD2*** | **XM_005682735.1** | **-0,85** | **0,56** | **DTW domain containing 2** |
| ***EHHADH*** | **XM_005675175.1** | **0,51** | **1,42** | **enoyl-CoA, hydratase/3-hydroxyacyl CoA dehydrogenase** |
| ***EPB41L3*** | **XM_005697125.1** | **0,70** | **1,62** | **erythrocyte membrane protein band 4.1-like 3** |
| ***EPSTI1*** | **XM_005687584.1** | **0,64** | **1,56** | **epithelial stromal interaction 1 (breast)** |
| ***ESPN*** | **XM_005690834.1** | **-0,64** | **0,64** | **espin** |
| ***F2R*** | **XM_005685292.1** | **-0,74** | **0,60** | **coagulation factor II (thrombin) receptor** |
| ***FCRL2*** | **XM_005677296.1** | **0,63** | **1,54** | **Fc receptor-like 2** |
| ***FMNL2*** | **XM_005676239.1** | **0,78** | **1,72** | **formin-like 2** |
| ***FMO2*** | **XM_005690635.1** | **1,11** | **2,16** | **flavin containing monooxygenase 2 (non-functional)** |
| ***FRMD4B*** | **XM_005695748.1** | **0,76** | **1,69** | **FERM domain containing 4B** |
| ***GZMM*** | **XM_005682880.1** | **-0,78** | **0,58** | **granzyme M (lymphocyte met-ase 1)** |
| ***HERC5*** | **XM_005681669.1** | **0,54** | **1,46** | **HECT and RLD domain containing E3 ubiquitin protein ligase 5** |
| ***HTRA1*** | **XM_005698569.1** | **-0,57** | **0,67** | **HtrA serine peptidase 1** |
| ***IFI44*** | **XM_005678196.1** | **0,83** | **1,78** | **interferon-induced protein 44, transcript variant X2** |
| ***IFI44*** | **XM_005678197.1** | **0,73** | **1,66** | **interferon-induced protein 44, transcript variant X3** |
| ***IFI44L*** | **XM_005678249.1** | **0,75** | **1,68** | **interferon-induced protein 44-like** |
| ***IFI6*** | **XM_005676790.1** | **1,11** | **2,16** | **interferon, alpha-inducible protein 6** |
| ***IFIT1*** | **XM_005698194.1** | **0,99** | **1,99** | **interferon-induced protein with tetratricopeptide repeats 1** |
| ***IFIT3*** | **XM_005698195.1** | **1,06** | **2,09** | **interferon-induced protein with tetratricopeptide repeats 3, transcript variant X1** |
| ***IFIT3*** | **XM_005698196.1** | **0,68** | **1,60** | **interferon-induced protein with tetratricopeptide repeats 3, transcript variant X2** |
| ***IFIT5*** | **XM_005698239.1** | **0,73** | **1,66** | **interferon-induced protein with tetratricopeptide repeats 5** |
| ***IMPG2*** | **XM_005674860.1** | **-0,87** | **0,55** | **interphotoreceptor matrix proteoglycan 2** |
| ***ISG15*** | **XM_005690795.1** | **1,69** | **3,23** | **ISG15 ubiquitin-like modifier** |
| ***KIAA1324*** | **XM_005677961.1** | **0,51** | **1,43** | **KIAA1324 ortholog, transcript variant X1** |
| ***KLRF1*** | **XM_005680867.1** | **-1,05** | **0,48** | **killer cell lectin-like receptor subfamily F, member 1** |
| ***KLRK1*** | **XM_005680842.1** | **-0,50** | **0,71** | **killer cell lectin-like receptor subfamily K, member 1** |
| ***LAMP3*** | **XM_005675214.1** | **0,59** | **1,51** | **lysosomal-associated membrane protein 3, transcript variant X1** |
| *HA25* | XM_005701684.1 | 1,24 | 2,36 | HA25 (LOC100860813) |
| *GPR68* | XM_005702029.1 | -0,59 | 0,67 | ovarian cancer G-protein coupled receptor 1-like (LOC102168821) |
| *CCL5* | XM_005699389.1 | 0,80 | 1,74 | c-C motif chemokine 5-like (LOC102169556) |
|  | XM_005675140.1 | 0,83 | 1,78 | uncharacterized LOC102170912 |
| *FADS2* | XM_005699818.1 | -0,97 | 0,51 | fatty acid desaturase 2-like (LOC102171133) |
| *APOL3* | XM_005701671.1 | -0,63 | 0,65 | apolipoprotein L3-like (LOC102171143) |
|  | XR_311005.1 | 1,21 | 2,32 | uncharacterized LOC102171392 |
| *TPCN1* | XM_005686411.1 | 0,81 | 1,75 | two pore calcium channel protein 1-like (LOC102171434) |
| *LYZ1* | XM_005680192.1 | -0,71 | 0,61 | lysozyme C-1-like (LOC102172037) |
|  | XM_005701454.1 | 0,86 | 1,82 | SLA class II histocompatibility antigen, DQ haplotype D alpha chain-like (LOC102172887) |
|  | XM_005701898.1 | -0,73 | 0,60 | antigen WC1.1-like (LOC102174561) |
| *ZNF347* | XM_005692737.1 | 0,59 | 1,51 | zinc finger protein 347-like (LOC102174966) |
| *HBBC* | XM_005689813.1 | -1,60 | 0,33 | hemoglobin subunit beta-C-like (LOC102175045) |
|  | XM_005701422.1 | -0,89 | 0,54 | BOLA class I histocompatibility antigen, alpha chain BL3-6-like (LOC102176782) |
|  | XM_005701685.1 | 0,86 | 1,81 | boLa class II histocompatibility  antigen, DQB*0101 beta chain-like (LOC102176786) |
| *EMR3* | XM_005682432.1 | -0,93 | 0,52 | EGF-like module-containing mucin-like hormone receptor-like 3-like (LOC102178529) |
| *C4BPA* | XM_005690428.1 | 0,68 | 1,60 | C4b-binding protein alpha chain-like (LOC102179403) |
| *PRSS2* | XM_005679544.1 | -0,74 | 0,60 | anionic trypsin-like (LOC102179545) |
| *MYADM* | XM_005695019.1 | -1,05 | 0,48 | myeloid-associated differentiation marker-like (LOC102179985) |
| *USP41* | XM_005680768.1 | 0,70 | 1,63 | putative ubiquitin carboxyl-terminal hydrolase 41-like (LOC102180290) |
|  | XM_005691749.1 | 0,52 | 1,43 | uncharacterized LOC102180790 |
| *MRP4* | XM_005701314.1 | -1,76 | 0,29 | multidrug resistance-associated protein 4-like (LOC102181111) |
|  | XM_005701539.1 | -0,87 | 0,55 | BOLA class I histocompatibility antigen, alpha chain BL3-7-like (LOC102182025) |
|  | XM_005701270.1 | -0,78 | 0,58 | uncharacterized LOC102183501 |
|  | XM_005701814.1 | -0,66 | 0,63 | antigen WC1.1-like (LOC102183687) |
|  | XM_005696678.1 | -0,92 | 0,53 | BOLA class I histocompatibility antigen, alpha chain BL3-7-like (LOC102184105) |
| *KLRD1* | XM_005680843.1 | -0,66 | 0,63 | natural killer cells antigen CD94-like (LOC102184229) |
| *MRP4* | XM_005701542.1 | -1,16 | 0,45 | multidrug resistance-associated protein 4-like (LOC102184240) |
| *SERPINB3* | XM_005697328.1 | -0,81 | 0,57 | serpin B3-like (LOC102184299) |
| *OAS1* | XM_005701870.1 | 1,70 | 3,24 | 2'-5'-oligoadenylate synthase 1-like (LOC102185558) |
|  | XM_005701959.1 | -1,46 | 0,36 | BOLA class I histocompatibility antigen, alpha chain BL3-7-like (LOC102185917) |
|  | XR_311067.1 | -1,85 | 0,28 | uncharacterized LOC102186545, transcript variant X1 |
| *LGALS9* | XM_005701701.1 | 1,22 | 2,33 | galectin-9-like (LOC102186681) |
| *DD3* | XM_005701156.1 | -0,93 | 0,52 | dihydrodiol dehydrogenase 3-like (LOC102187204), transcript variant X1 |
| *SIGLEC14* | XM_005701965.1 | 1,06 | 2,09 | sialic acid-binding Ig-like lectin 14-like (LOC102188938) |
|  | XM_005701559.1 | -0,95 | 0,52 | antigen WC1.1-like (LOC102190214) |
|  | XM_005696530.1 | -0,64 | 0,64 | DLA class II histocompatibility antigen, DR-1 beta chain-like (LOC102190745) |
| *METAP2* | XM_005700899.1 | 0,57 | 1,49 | methionine aminopeptidase 2-like (LOC102190867) |
| *OAS1* | XM_005691488.1 | 1,42 | 2,68 | 2'-5'-oligoadenylate synthase 1-like (LOC102190983), transcript variant X2 |
| ***LY6E*** | **XM_005688801.1** | **0,70** | **1,62** | **lymphocyte antigen 6 complex, locus E (LY6E)** |
| ***MARK1*** | **XM_005690579.1** | **0,58** | **1,50** | **MAP/microtubule affinity-regulating kinase 1 (MARK1)** |
| ***MX1*** | **XM_005675747.1** | **1,09** | **2,13** | **myxovirus (influenza virus) resistance 1, interferon-inducible protein p78 (mouse) (MX1)** |
| ***MX2*** | **XM_005675746.1** | **1,50** | **2,82** | **myxovirus (influenza virus) resistance 2 (mouse) (MX2)** |
| ***NOMO1*** | **XM_005697968.1** | **0,54** | **1,45** | **NODAL modulator 1 (NOMO1)** |
| ***NT5E*** | **XM_005684823.1** | **-0,98** | **0,51** | **5'-nucleotidase, ecto (CD73) (NT5E)** |
| ***OAS1*** | **XM_005709622.1** | **1,68** | **3,20** | **2'-5'-oligoadenylate synthetase 1, 40/46kDa (OAS1)** |
| ***OAS3*** | **XM_005709604.1** | **1,65** | **3,13** | **2'-5'-oligoadenylate synthetase 3, 100kDa (OAS3)** |
| ***PARPBP*** | **XM_005680530.1** | **-0,62** | **0,65** | **PARP1 binding protein** |
| ***PCOLCE*** | **XM_005697822.1** | **-0,55** | **0,68** | **procollagen C-endopeptidase enhancer** |
| ***PIGR*** | **XM_005690418.1** | **-0,84** | **0,56** | **polymeric immunoglobulin receptor** |
| ***PLAU*** | **XM_005699221.1** | **0,57** | **1,48** | **plasminogen activator, urokinase** |
| ***PRF1*** | **XM_005699151.1** | **-0,61** | **0,65** | **perforin 1 (pore forming protein)** |
| ***PRNP*** | **XM_005688157.1** | **-3,68** | **0,08** | **prion protein** |
| ***RHCG*** | **XM_005694970.1** | **-1,95** | **0,26** | **Rh family, C glycoprotein** |
| ***RPL35A*** | **XM_005675050.1** | **0,65** | **1,57** | **ribosomal protein L35a, transcript variant X2** |
| ***SEZ6L*** | **XM_005691699.1** | **1,90** | **3,73** | **seizure related 6 homolog (mouse)-like (SEZ6L)** |
| ***SIGLEC1*** | **XM_005688201.1** | **0,74** | **1,67** | **sialic acid binding Ig-like lectin 1, sialoadhesin** |
| ***SIGLEC14*** | **XM_005692896.1** | **0,84** | **1,79** | **sialic acid binding Ig-like lectin 14** |
| ***SLC27A5*** | **XM_005693070.1** | **-1,81** | **0,29** | **solute carrier family 27 (fatty acid transporter) member 5** |
| ***STS*** | **XM_005701386.1** | **-0,77** | **0,59** | **steroid sulfatase (microsomal), isozyme S** |
| ***SULT1C4*** | **XM_005686652.1** | **-0,54** | **0,69** | **sulfotransferase family, cytosolic, 1C, member 4** |
| ***TBC1D16*** | **XM_005694405.1** | **0,55** | **1,47** | **TBC1 domain family, member 16** |
| ***TFCP2L1*** | **XM_005676324.1** | **-0,73** | **0,60** | **transcription factor CP2-like 1** |
| ***TIGIT*** | **XM_005674945.1** | **-0,59** | **0,67** | **T cell immunoreceptor with Ig and ITIM domains** |
| ***TJP3*** | **XM_005682657.1** | **-0,61** | **0,66** | **tight junction protein 3** |
| ***TRPV4*** | **XM_005691683.1** | **0,90** | **1,87** | **transient receptor potential cation channel, subfamily V, member 4** |
| ***TXNDC5*** | **XM_005696892.1** | **-0,53** | **0,69** | **thioredoxin domain containing 5 (endoplasmic reticulum)** |
| ***XAF1*** | **XM_005693425.1** | **0,53** | **1,45** | **XIAP associated factor 1, transcript variant X2** |

*Annotated genes are marked with bold letters
